# *BRAF*^V600E^ mutation and DSS treatment synergize to induce cecal tumor formation in mice

**DOI:** 10.1016/j.bbrep.2023.101634

**Published:** 2024-01-01

**Authors:** Chenxi Gao, Farzad Esni, Edward Chu, Jing Hu

**Affiliations:** a Division of Gastroenterology, Hepatology and Nutrition, Department of Medicine, University of Pittsburgh School of Medicine, Pittsburgh, PA, 15213, USA; b UPMC Hillman Cancer Center, University of Pittsburgh School of Medicine, Pittsburgh, PA, 15213, USA; c Department of Surgery, University of Pittsburgh School of Medicine, Pittsburgh, PA, 15213, USA; d Albert Einstein Cancer Center, Albert Einstein College of Medicine, Bronx, NY, 10461, USA

**Keywords:** *BRAF*^V600E^ mutation, DSS, CRC

## Abstract

*BRAF* mutation is a driver mutation in colorectal cancer (CRC), and *BRAF*^V600E^ mutation is found in 10–15 % of all CRCs. *BRAF* mutant CRCs in patients are primarily localized in the right colon, including the cecum. However, in the *Vill-Cre*;*BRAF*^V600E/+^ mice, adenomas mainly developed in the small intestines of the mice, and no tumor formed in the cecum. The mice model of *BRAF*^V600E^-mutant CRC with tumors in the cecum is lacking. Dextran Sulfate Sodium (DSS) treatment induces colitis in mice. Acute DSS treatment does not lead to tumor formation. We show that DSS treatment and BRAF^V600E^ mutation synergistically induced cecal tumorigenesis, and cecal tumors formed within three months after five-day DSS treatment. The location of the adenomas supports the patient relevance of the model. Our BRAF^V600E^/DSS model provides a valuable *in vivo* model for future identification and validation of novel therapeutic approaches for treating *BRAF*-mutant CRC. Our results are consistent with the notion that *BRAF*^V600E^ mutation is an oncogenic event that can shift controlled regeneration to unrestrained oncogenesis.

## Highlights

• Dextran Sulfate Sodium (DSS) treatment induces colitis in mice. Acute DSS treatment does not lead to tumor formation.
• In the *Vill-Cre*; *BRAF*^V600E/+^ mice, no tumor developed in the cecum.
• We found that five-day DSS treatment and BRAF^V600E^ mutation synergistically induced cecal tumor formation in mice.
• *BRAF*^V600E^ mutation is an oncogenic event that shifts controlled regeneration to unrestrained oncogenesis.

## Graphical abstract

**Figure:**
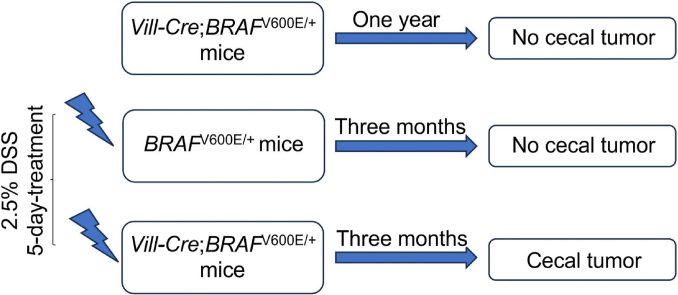

## 1 Introduction

About 10–15 % of CRC patients are characterized by a mutation in the B-Raf proto-oncogene serine/threonine kinase (BRAF) gene [1]. In CRC, the majority (80%–90 %) of mutations in BRAF are V600E (valine to glutamic acid mutation) [2]. *BRAF*-mutant CRCs are primarily located in the right colon, including the cecum [3]. However, in *Vill-Cre*;*Braf*^V600E/+^ mice, adenomas primarily developed in the small intestines at ages older than ten months, and no tumors developed in the cecum [4]. It has been shown that right-sided tumorigenesis is supported by microbial-driven inflammation [5]. This study explored whether dextran sulfate sodium (DSS)-induced inflammation plays a role in *BRAF*^V600E^-induced tumorigenesis in mice.

DSS is a water-soluble, negatively charged sulfated polysaccharide. DSS treatment in mice induces intestinal tissue damage (colitis) [6,7]. The mechanism by which DSS causes intestinal inflammation is unclear but is likely the result of damage to the epithelial monolayer lining the large intestine, allowing the dissemination of proinflammatory intestinal contents (e.g., bacteria and their products) into underlying tissue. Notably, 5 or 7 days of DSS treatment does not induce adenoma formation in mice intestines. To test whether DSS-induced inflammation impacts genetic mutation (e.g., BRAF^V600E^ mutation)-induced tumorigenesis, we compared the phenotypic consequences of DSS treatment in control mice and *Vill-Cre*;*BRAF*^V600E/+^ mice. Our results revealed that DSS treatment and *BRAF*^V600E^ synergistically induced cecal tumor formation in mice.

## 2 Material and methods

### 2.1 Mice and treatment

All animal procedures were performed according to protocols approved by the Institutional Animal Care and Use Committee at the University of Pittsburgh. Mice were fed a standard diet (diet ID 5P75; Purina LabDiet). *Villin-Cre* (cat. no. 021504), *BRAF*^*LSL-V600E/+*^ (cat. no. 017837), and *Rosa26-tdTomato* (cat. no. 007914) mice were obtained from the Jackson Laboratory. *Villin-Cre* and *BRAF*^*LSL-V600E/+*^ mice were crossed to get the *Vill-Cre*;*BRAF*^V600E/+^ (*BC*) mice. The littermates harboring the *BRAF*^*LSL-V600E*^ allele were used as controls whenever available. *Villin-Cre* mice and *Rosa26*^*LSL-tdTomato/LSL-tdTomato*^ mice were crossed to get the *Villin-Cre*; *Rosa26*^*LSL-tdTomato/+*^ mice. Genotyping was performed according to the protocols provided by the Jackson Laboratory. Dextran Sulfate Sodium (DSS) was purchased from Fisher Scientific (molecular weight 40000–50000 kDa). To study DSS-induced tumors, the 1.5-month-old *BC* mice were treated with 2.5 % (w/v) DSS for five days. Then, the DSS solution was withdrawn, and the mice were given regular drinking water. The tumor formation was evaluated three months or 8.5–10.5 months after the treatment. *BRAF*^*LSL-V600E/+*^ mice treated with DSS were used as controls.

### *2.2* In situ hybridization

In situ hybridization (ISH) was performed using the Advanced Cell Diagnostics RNAscope 2.5 HD Reagent Kit-BROWN (cat. no. 322300) according to the manufacturer's instructions. The following probes from Advanced Cell Diagnostics were used: *Lgr5* (cat. no. 312171), *Lgr4* (cat. no. 318321), *Axin2* (cat. no. 400331).

### 2.3 Hematoxylin and eosin (H&E) staining and immunohistochemistry (IHC) staining

After the mice were euthanized, the cecum was dissected out, rinsed twice with ice-cold PBS, fixed overnight in 10 % neutral buffered formalin, embedded in paraffin, and finally cut into 5-μm sections. The sections were deparaffinized in xylenes and rehydrated in graded alcohol solutions, followed by washes in distilled water. Then the sections were stained by hematoxylin (Fisher Scientific, cat. no. 50-261-10), eosin (Fisher Scientific, cat. no. NC0236648), dehydrated, and coverslipped with Epredia Cytoseal XYL Mountant (Fisher Scientific, cat. no. 22-050-262).

The IHC staining with anti–β-catenin antibody was performed as previously described [8]. The sections were deparaffinized and rehydrated, as mentioned above. The antigen retrieval was performed for 30 min in 10 mmol/L sodium citrate (pH 6) solution supplemented with 0.05 % tween-20. After washing with phosphate-buffered saline with tween-20 (PBST) buffer, the endogenous peroxidase was blocked with 3 % hydrogen peroxide for 10 min, followed by blocking with 20 % goat serum diluted in PBS for 30 min. Sections then were incubated with anti–β-catenin antibody (Cell Signaling Technology, cat. no. 9582) diluted in 10 % goat serum in PBS at room temperature for 2 h. The sections were washed again with PBST and incubated with HRP polymer anti-rabbit IgG reagent (Vector Laboratories, cat. no. MP-7801-15) for 1 h at room temperature. Color visualization was performed with 3.3′-diaminobenzidine. The sections were counterstained with hematoxylin, dehydrated, and coverslipped with permanent mounting media. For IHC staining with anti-RFP antibody (Rockland Immunochemicals, cat. no. 600-401-379), the procedures were modified slightly, as follows: (1) antigen retrieval was performed for 10 min in sodium citrate buffer (pH 6); (2) all wash steps were performed with PBS buffer; and (3) primary antibody incubation was performed overnight at 4 °C.

The H&E, IHC, and ISH staining shown in the figures are representative results from at least three mice.

### 2.4 MSI analysis

The DNA was extracted from FFPE tissue sections using QIAamp DNA FFPE Tissue Kit (Qiagen, cat. no. 56404). Cecal tissues of 6-week-old *C5*7BL*/6* mice were used as control. According to a prior report [4], five microsatellite repeat markers, Bat24, Bat26, Bat30, Bat37, and Bat64, were used for MSI analysis. PCR amplification was carried out in a multiplex reaction using HSTaq polymerase (Takara Bio, Japan) with primer concentrations of 0.5 μM. The thermal cycling conditions were as follows: initial denaturation at 95 °C for 5 min, followed by 35 cycles of 95 °C for 30 s, 60 °C for 30 s, and 72 °C for 30 s; then a final extension step at 68 °C for 30 min. PCR fragments were analyzed by capillary electrophoresis, ABI3130XL (Life Technologies), and the GeneMapper ID3.2 program (Life Technologies). Tumor samples with greater or equal to 40 % MSI were classified as MSI-high (MSI-H), less than 40 % as MSI-low (MSI-L), and samples without alterations were classified as MSS.

## 3 Results and discussion

**Fig. 1:**
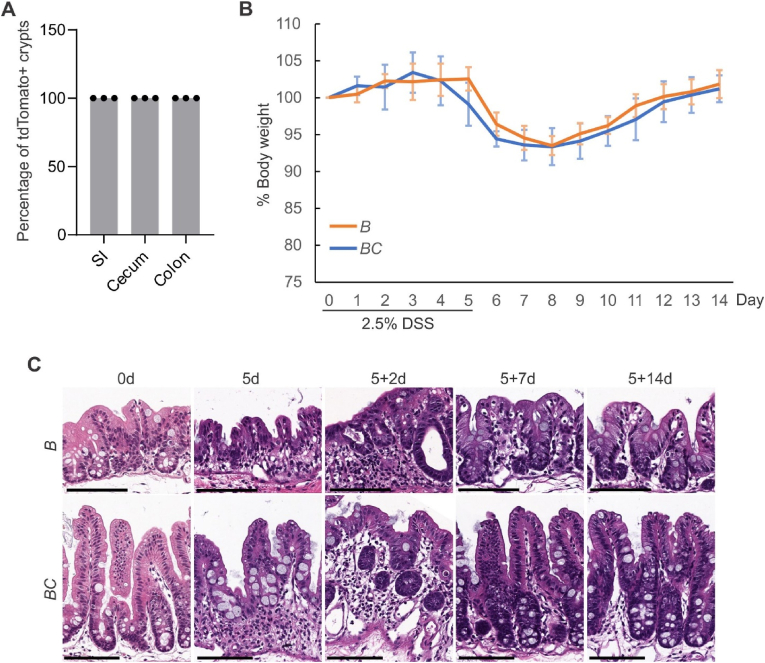
DSS treatment induced similar body weight change, tissue damage, and repairment in control and *BC* mice. (A) Cre-mediated recombination efficiency in *Villin-Cre*; *Rosa26*^*LSL-tdTomato/+*^ mice were scored for 30 crypts at each indicated bowel subsites (n = 3). **(B)** Body weight analysis of *BC* mice and control mice treated with 2.5 % DSS for 5 days and given regular drinking water thereafter. *BC* mice: n = 7; *B* mice: n = 6. Data presented as mean ± SD. *B* mice: *BRAF*^V600E/+^ mice. *BC* mice: *Vill-Cre*;*BRAF*^V600E/+^ mice. **(C)** Representative H&E staining of cecum from control and *BC* mice with indicated DSS treatment time and recovery time.

We treated the 6-week-old control *BRAF*^V600E/+^ mice and *Vill-Cre*;*BRAF*^V600E/+^ mice (male and female) with DSS (2.5 %) in drinking water for five days and then sacrificed the mice three months after the treatment. The 100 % Cre recombination efficiency indirectly validated the expression of BRAF^V600E^ in *Vill-Cre*;*BRAF*^V600E/+^ mice intestine (Fig. 1A). DSS treatment did not cause different patterns of body weight changes in these two groups of mice (Fig. 1B). From an H&E staining point of view, DSS-treatment-induced tissue damage and repairment were similar in these two groups (Fig. 1C). Together, these results implied that *BRAF*^V600E^ mutation does not affect DSS treatment-induced tissue damage and repairment.

**Fig. 2:**
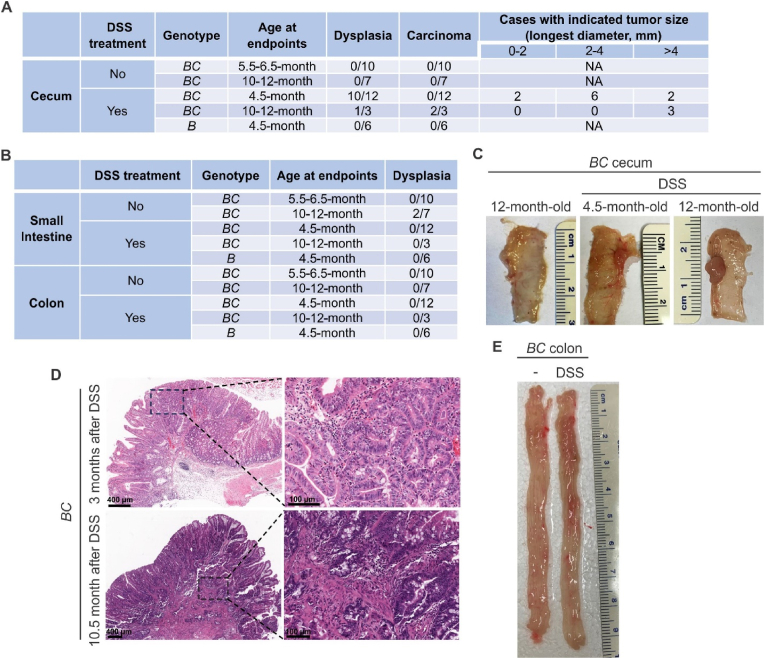
*BRAF*^V600E^ mutation and DSS treatment induce cecal tumor formation in mice. *BC* and control *B* mice at the age of 1.5 months were treated with 2.5 % DSS in drinking water for five days and then were given regular water. The mice were sacrificed 3 months or 8.5–10.5 months after DSS treatment. Summary of cecal tumor incidence **(A)** and small intestine and colon tumor incidence **(B)** in indicated mice at indicated age. *B* mice: *BRAF*^V600E/+^ mice. *BC* mice: *Vill-Cre*;*BRAF*^V600E/+^ mice. **(C)** Representative images of cecal tumors from indicated *BC* mice. **(D)** Representative hematoxylin and eosin (H&E) staining of cecal tumors from DSS-treated *BC* mice was shown. **(E)** Representative examples of the colon from 12-month-old untreated *BC* mice and DSS-treated *BC* mice.

However, despite the similarities in tissue damage and repairment, DSS treatment did synergize with *BRAF*^V600E^ mutation to induce cecal tumor formation. The cecal tumor incidence in DSS-treated *Vill-Cre*;*BRAF*^V600E/+^ mice (male and female) was 83.3 % (10/12) in 3 months after DSS treatment and 100 % (3/3) in 8.5–10.5 months after DSS treatment group (Fig. 2A, B and C). In contrast, no cecal tumor was found in DSS-treated *BRAF*^V600E/+^ mice. The H&E staining showed that the cecal tumors were adenomas three months after the DSS treatment group, and most tumors in 8.5–10.5 months after the DSS treatment group were carcinomas (Fig. 2D). DSS treatment appeared to have no impact on the appearance of the colon (Fig. 2E).

**Fig. 3:**
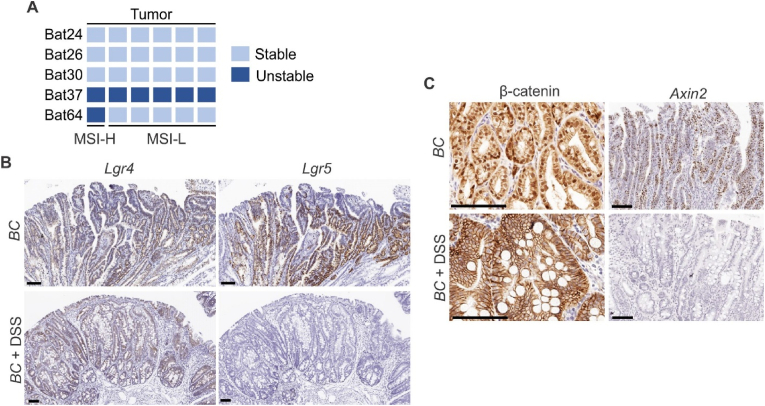
Molecular characterization of the cecal tumors. (A) Microsatellite instability status of cecal tumor from DSS-treated *BC* mice. Each column represents one sample. **(B)** and **(C)** Representative in situ hybridization (ISH) staining and immunohistochemistry (IHC) staining of tumor sections from the small intestine of *BC* mice and cecum of DSS-treated *BC* mice using anti-β-catenin antibody or indicated probes. Scale bars: 100 μm.

About 50 % of *BRAF*-mutated CRCs exhibit defective DNA mismatch repair [2]. The results of microsatellite instability (MSI) analysis indicated that the cecal tumors were microsatellite stable (MSI) (Fig. 3A). It has been shown that chemical injury induced by DSS leads to the loss of *Lgr5*+ cells and regeneration requires reprograming *Lgr4*+ differentiated cells [9]. We then performed in situ hybridization (ISH) to evaluate the expression pattern of *Lgr5* and *Lgr4* in the tumors. Unlike the tumors in the small intestine in *Vill-Cre*;*BRAF*^V600E/+^ mice that expressed both *Lgr5* and *Lgr4*, cecal tumor in DSS-treated *Vill-Cre*;*BRAF*^V600E/+^ mice primarily expressed *Lgr4* but not *Lgr5* (Fig. 3B). *Lgr5* is a Wnt/β-catenin pathway target. Consistent with the low expression of *Lgr5*, we found that while β-catenin in *BC* tumors was mostly nuclear, IHC results showed the membrane location of β-catenin in DSS treatment-induced tumors (Fig. 3C). The expression of *Axin2*, another transcriptional target of the Wnt/β-catenin pathway, was also inhibited in DSS-treatment-induced tumors (Fig. 3C). These data imply that DSS treatment-induced *BRAF*^V600E^-tumors were Wnt/β-catenin-low tumors.

Tissue regeneration and tumorigenesis share similar molecular pathways. While cancer is considered a continuous state of repair, a wound that does not heal [10], the determining factors that shift regeneration to carcinogenesis remain largely unknown. It has been suggested that during regeneration, oncogenic events (genetic or epigenetic) can lock the cell in an activated state of renewal, leading to tumor formation [11]. However, the nature of the oncogenic events in context-dependent regenerations remains largely unexplored. Our data suggest that BRAF mutation locks the intestinal epithelium in a state of repairment (high Lgr4 after DSS treatment).

Overall, our results described a novel *BRAF*^V600E^-mutant CRC model with cecal tumors. This model is valuable for future studies on *BRAF*-mutant CRC biology and for identifying and validating novel treatment approaches for BRAF-mutant CRCs.

## Funding

This investigation was supported in part by USPHS grant CA236965 to J Hu.

No potential conflicts of interest were disclosed.

## Ethics approval and consent to participate

Not applicable.

## Consent for publication

Not applicable.

## Availability of data and materials

Not appliable.

## CRediT authorship contribution statement

**Chenxi Gao:** Conceptualization, Data curation, Formal analysis, Investigation, Visualization, Writing – original draft. **Farzad Esni:** Formal analysis. **Edward Chu:** Formal analysis. **Jing Hu:** Conceptualization, Data curation, Formal analysis, Funding acquisition, Writing – original draft, Writing – review & editing.

## Declaration of generative AI and AI-assisted technologies in the writing process

Not appliable.

## Declaration of competing interest

None to declare.

## Data Availability

Data will be made available on request.
